# Clinic of Dr. William P. Johnston

**Published:** 1843-12-23

**Authors:** William P. Johnston

**Affiliations:** Professor of Surgery; Washington


### ABSTRACT OF TWO CLINICAL LECTURES,

*Delivered at the Dispensary of the Medical Department of the Columbian College, Washington, D. C.*

BY WILLIAM P. JOHNSTON, M. D.

Professor of Surgery.

Reported for the Medical Examiner.]

### AMAUROSIS.

*November 14th*, 1843 —Dr. J. commenced by saying that as amaurosis had been the subject of a recent clinic, he would not again describe the varieties of this disease with the treatment most appropriate to each particular form; he proposed on the present occasion to notice some of the peculiarities of the case before us, and to contrast the symptoms of this patient with those of the female, who presented herself at the clinic of last week. In examining the eyes of this patient the first thing that strikes our attention is the unnatural dilatation of both pupils, especially the left, and their inability to contract even when exposed to a bright light. Pursuing the examination a little further, you will remark within the pupil of the right eye, and apparently at the very bottom of this organ, a yellowish concave opacity or discolouration. In the left eye, a similar opacity is seen, more limited however in extent, for around it we recognise the natural black colour. Before examining into the history of the case, let us see whether we can arrive at a diagnosis by the aid alone of the data furnished us by the inspection of the eye. Can this be a case of cataract? Certainly not; for in the first place the opacity is deep within the cavity of the eye, and far beyond the lens. Let us now examine the eyes with a lighted candle, after the method of Sanson. I can now recognise distinctly, as in an healthy eye, the three images of the candle. The first, distinct and well defined, reflected by the cornea; the second more diffused, reflected by the anterior capsule of the lens; the third presents a well defined margin, but is an inverted image, owing to the posterior capsule of the lens being concave, and moves in a direction opposed to that of the other two, and of the light itself, as it is alternately elevated and depressed. It is now certain that the opacity is not in the lens, otherwise we should no longer recognise the second and third images, at least as distinctly as we have done in this case. As this is not a case of cataract, we think it must be one of amaurosis, from some organic lesion of the retina. Let us see if the history of the patient will support us in this opinion. We are told by the patient that he has been going blind for about two years; formerly he was much annoyed by seeing, as he says, "stars," or bright specks, which appeared to float before his eyes. These are now no longer visible, but his sight has become still more impaired; objects are now imperfectly seen, they are ill defined and confused. We have now sufficient to warrant a positive diagnosis; permanently dilated pupils; a concave opacity at the bottom of the eye; abolition of sight almost complete, succeeding to altered or deranged function of the organ, during which strange and unnatural objects were perceived. There is something very striking in the exterior appearance of an amaurotic patient. I noticed it in the man before us, and it first awakened my suspicions as to the nature of his case. He walks with his head elevated, his eyes steadily gazing before him; if vision be not entirely lost you will see him selecting the sunny side of the street or turning his eyes towards the point from which the brightest light proceeds. In these cases of torpid amaurosis the retina becomes more and more insensible as the sight declines until at last the patient is enabled to gaze at the sun with impunity.

There are one or two circumstances connected with the present state of this patient to which I must now call your attention. In the first place he has told us that when he wishes to see an object he does not look directly at it, but laterally; this is easily explained. We have seen that in the right eye, in which the sight is less impaired than in the left, the opacity seen through the pupil occupies the centre, while around we perceive the normal black colour. Now if the patient looks directly at an object, the image must impinge upon the centre of the retina, or upon the part most diseased; whereas, if he regards it obliquely, the image falls to one side, or upon a portion of the retina which is less affected. In a similar manner we must account for another peculiarity in this case. We mentioned that in torpid or organic amaurosis, the patient discerns the light better at mid-day, or in a very bright light; the patient before us, on the contrary, has said that he sees better towards dark. This is not owing to photophobia, produced by irritation or inflammation of the retina, because none such exists; but rather to the fact that towards dusk the pupil of the right eye, which at this time you find more contracted than the opposite one, will dilate to a moderate extent and allow the image of an object to be reflected more readily upon some portion of the retina beyond the opacity. The retina is not always affected throughout its whole extent in amaurosis, as the present case shows; sometimes a single point is diseased and the patient sees a black spot always before his eyes. The function of vision may become deranged in various ways; sometimes the patient sees double; a bright light or halo may surround an object; sparks or flashes of light may be seen, or spots may be constantly floating before the eyes (muscæ volitantes;) objects may appear disfigured, or only a portion of the same may be seen. A young lady of nervous temperament whom I attended about a year since, had been suffering for some days with a dull headache, when suddenly, while intently occupied in some worsted work, she raised her eyes declaring that she could see only the superior half of whatever she gazed at. This partial amaurosis soon subsided; violent headache, morbid acuteness of hearing, and a strong bounding pulse followed; it was some days before these symptoms subsided.

To return: the examination that we have made of our patient, has led us to the conclusion that this case is one of amaurotic ambliopia, which has already nearly terminated in torpid amaurosis, the result of some organic lesion of the retina.

Prognosis most unfavourable; the patient has already been under the hands of physicians, both in Virginia and in Philadelphia, and finds himself in no way benefited. Sooner or later the imperfect vision which is left to him will be entirely lost.

Before dismissing this case let me again remind you of the one we saw last week. This latter was an example of irritable amaurosis, complicated as it often is, with congestion of the retina. I allude to this case that you may contrast the pupils of the patient before us with those of the former. In this man the pupils are permanently dilated but perfectly round. In the female that we saw last week the pupil of the affected eye was contracted and of an irregular shape; the irritation had besides extended from the eye to the lids, and hence the constant batting of the lids to which I called your attention.

LENTICULAR CATARACT, (HARD.)

*November 17th.*—Betsey Butler, æt. 75 years, of good constitution, has enjoyed an unusual share of health. About four years since she first began to notice that her sight was becoming impaired; objects appeared to her less distinct than natural, and as if covered by a veil. She has never received any injury about the head, nor has she suffered from pain in the eyes or head. She long since remarked that she could see more distinctly towards evening, or when her back was turned to the light; in the day, or when gazing at a bright light, she could see but little. Her sight has continued to diminish little by little; about a year since she ceased to see by the left eye. At this moment she is still enabled to see somewhat with the right eye, sufficiently to recognise objects held before her.

If we examine the right eye we shall find that the iris appears healthy, it dilates and contracts readily, it is free from every anormal adhesion, and the pupil is perfectly round. If we look through the pupil, we find at a short distance behind, a yellowish green opacity of a dull colour; around the margin of this opacity we perceive a black rim, being the shadow cast by the iris upon the opaque lens, (this may also in part be due to the uvea which forms the edge of the pupil.) The left eye presented a similar appearance, except that the opacity was more marked. We performed the operation of couching upon this eye about a month since, we shall see with what success.

How are we to know that this case is one of cataract, and not some other affection? It would be impossible to confound this case with any but glaucoma or amaurosis. In glaucoma the opacity offers some shade of green; thus far there is a resemblance between this disease and the one before us, in which, as we have said, the opacity is of a yellowish green. But in glaucoma the opacity is deep within the eye, it is concave and cannot be seen when the eye is viewed laterally; the contrary we have seen obtains in the patient before us. Again, in hard lenticular cataract, of which this is an example, the shadow cast by the iris upon the opacity behind, is a valuable sign which is never seen in glaucoma.

Cataract can never be mistaken for confirmed amaurosis. In the latter disease the pupils are widely and permanently dilated and vision is either entirely abolished or the sensibility of the retina can only be awakened by the brightest light. In cataract, on the contrary, the pupil dilates and contracts readily, and as the opaque lens offers a mechanical obstruction to the passage of the rays of light, the patient is compelled to seek obscurity, to shade his eyes or turn his back towards the window in order that the pupil may dilate, and the rays of light then be admitted through portions of the lens most distant from the centre; the opacity in these cases of hard lenticular cataract being most marked in the centre, and diminishing from this point to the circumference.

We have still another method of distinguishing cataract from both glaucoma and amaurosis; we allude to the signs furnished by the catoptric method of examination. At our last lecture, we noticed that when a light was held to the eye of the patient with confirmed amaurosis, the three images of the candle were seen as distinctly as in an healthy eye. In lenticular cataract, on the contrary, the third or inverted image reflected by the posterior capsule, loses its distinctness at the commencement of the opacity, and when the cataract becomes fully formed or matured this image is no longer to be seen. The second erect image becomes also indistinct. In the right eye of the patient, whose case we are now considering, you will see on close examination that the second and third images of the candle are barely perceptible, if at all. We have now satisfactorily shown that our patient is affected with lenticular cataract; this cataract is moreover of firm consistence, which is indicated by its dark colour, and the distance at which it is seen from the pupil, increasing the dimensions of the posterior chamber, the latter being the result of the contraction of the lens, and finally by the opacity being more complete in the centre than towards the circumference.

When we first saw this case we at once decided upon the propriety of an operation. The patient had lost the sight of one eye entirely, and that of the other was much impaired. By dilating the pupil of the eye in which the cataract was completely matured, we found that she could then recognise objects around her; it was clear therefore that the case was not complicated with amaurosis. The patient was advanced in years, (75,) but age alone does not constitute a contra-indication to the operation. Her constitution was good, her general health perfect; besides this she was of a spare habit of body, all of which was in her favour. The operation of couching was performed about a month since; we were careful in the operation, to avoid carrying the lens so low as to press upon the retina, by which we might have produced incurable amaurosis. The patient suffered but little during the operation, a most favourable sign, as very acute inflammation never follows under such circumstances. There was no after treatment instituted except the administration of a few drops of laudanum and low diet. The eye was examined daily after the second day; no local inflammation supervened, but slight pain was felt in the eye, and there was no constitutional reaction. The cure has been perfect; the patient can now readily recognise the hands of a watch; with the aid of a suitable pair of glasses, her sight will still improve.

You remark that only one eye was operated upon; the reason for this was that the patient could still see by the other eye, and as there is always more or less risk attending the operation, it was more prudent not to endanger the eye which was still useful to her. In time the cataract of the right eye will also become mature, and should any accident happen to the eye, upon which we have already operated, it will then be time enough to remove the lens from behind the pupil of the other.

The operation of extraction is equally applicable to cases of hard cataract; couching, however, can be performed with greater facility, and it has also this great advantage over the former, that it may be repeated a second or even a third time if necessary. When the eye is deep within the socket, which is the case with this patient, couching should always be preferred; this latter operation has also been recommended in very aged persons who have the "arcus senilis" well marked.

*Washington, Nov. 20th,* 1843.

